# Heterotaxy syndrome with biliary atresia: a case report

**DOI:** 10.11604/pamj.2025.51.4.47028

**Published:** 2025-05-06

**Authors:** Amal Irfan Khazi, Mohga Yasser, Dafalla Rahamtalla, Bhavna Gupta

**Affiliations:** 1 Pediatric Medicine, Al Qassimi Women’s and Children’s Hospital, Sharjah, United Arab Emirates,; 2 Pediatric and Neonatology, Gastroenterology, Al Qassimi Women´s and Children´s Hospital (AQWCH), Emirates Health Services (EHS), Sharjah, United Arab Emirates,; 3 Pediatric and Neonatology, General Pediatrics, Al Qassimi Women´s and Children´s Hospital (AQWCH), Emirates Health Services (EHS), Sharjah, United Arab Emirates

**Keywords:** Case report, pediatric, heterotaxy syndrome, biliary atresia, heterotaxy syndrome with biliary atresia

## Abstract

We report the case of a late preterm female neonate, born at 36 weeks' gestation, with heterotaxy syndrome, severe congenital heart defects (dextrocardia, situs inversus, left atrial isomerism, complete atrioventricular septal defect (AVSD), and double outlet right ventricle), and biliary atresia. The association of biliary atresia with heterotaxy syndrome is exceptionally rare and adds complexity to the patient´s management. The patient underwent a laparoscopic Kasai procedure, which resulted in significant clinical improvement. This case highlights the importance of early diagnosis, a multidisciplinary approach, and timely surgical intervention in managing rare and complex conditions.

## Introduction

Heterotaxy syndrome is a complex disorder resulting from disruptions in the embryonic development of organ laterality, leading to the misplacement of thoracic and abdominal organs, and often accompanied by severe congenital heart defects [1,2]. While heterotaxy syndrome is primarily recognized for its cardiac and gastrointestinal anomalies, the association with biliary atresia is exceptionally rare, and it adds complexity to patient management and the importance of early diagnosis and a multidisciplinary approach [3,4]. We report a case of a late preterm neonate with heterotaxy syndrome, severe congenital heart defects, and biliary atresia, highlighting the challenges and management strategies for this rare association.

## Patient and observation

**Patient information:** our patient, a female neonate from the Philippines, was born premature at 36 weeks of gestation to non-consanguineous parents via vacuum-assisted vaginal delivery. Her Apgar score was 8 at 1 minute and 9 at 5 minutes. Her weight at birth was 2.57 kg. The mother, who was 37-years-old, had a history of gestational diabetes and pregnancy-induced hypertension, both of which were managed by medications. It is worth noting though, that her antenatal follow-ups were inconsistent. Immediately after birth, the patient was admitted to the neonatal intensive care unit (NICU) due to respiratory distress.

**Clinical findings:** on physical examination, she was alert, crying, and her skin was pink. No dysmorphic features were noted, but the patient was in visible respiratory distress, had labored breathing, and grunting on auscultation. A 4/6 systolic murmur was heard at the right upper sternal border. The abdomen was soft, non-tender, with the liver palpable 1cm below the costal margin, and normal female genitalia were noted.

**Timeline of current episode:** immediately after birth, the patient was admitted to the NICU due to respiratory distress. She was initially placed on continuous positive airway pressure (CPAP), but as oxygen saturation levels dropped, she was upgraded to biphasic positive airway pressure (BiPAP) and later intubated. On day 6 of life, the patient developed cholestatic jaundice, with yellowing of the skin and pale stools, prompting the initiation of treatment with ursodiol and double phototherapy. By day 16, the patient showed clinical improvement, was able to maintain oxygenation on room air, and was transferred to the general pediatric ward. Over the next few weeks, the patient progressed well, becoming clinically stable, passing yellow stools, tolerating formula milk every 3 hours, and maintaining oxygen saturation on room air.

**Figure 1:**
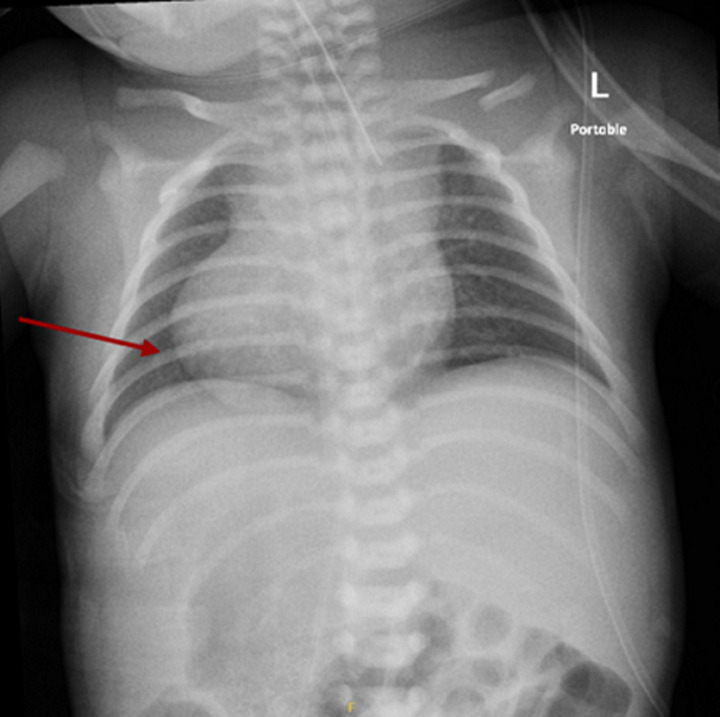
initial chest X-ray showed situs inversus, and clear lung fields; the red arrow points to dextrocardia; normal cardiothoracic ratio

**Figure 2:**
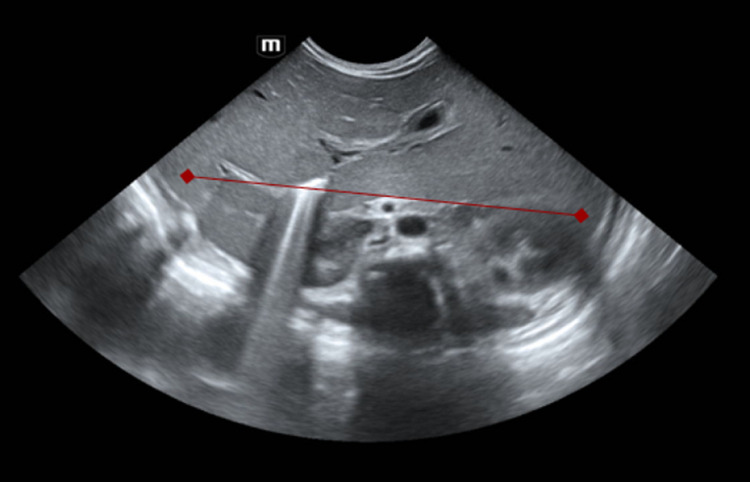
abdomen ultrasound shows the liver is enlarged occupying both hypochondria; no focal lesion and hepatic veins and inferior vena cava are not congested; portal vein is not seen dilated

**Figure 3:**
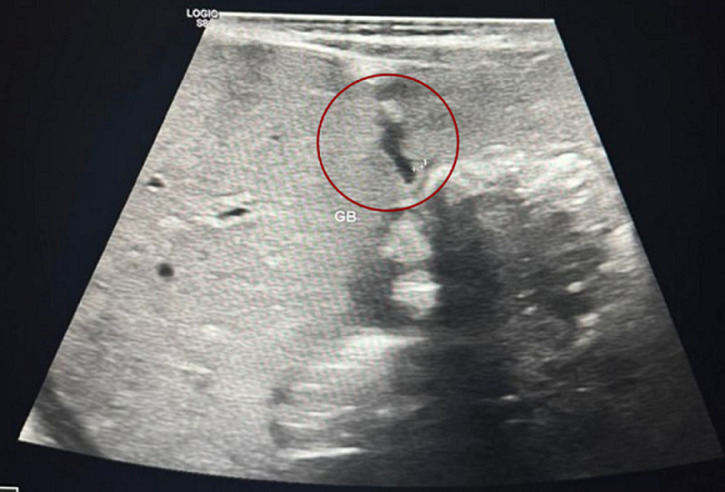
gallbladder seen on the left side, partially contracted during the fasting exam

**Figure 4:**
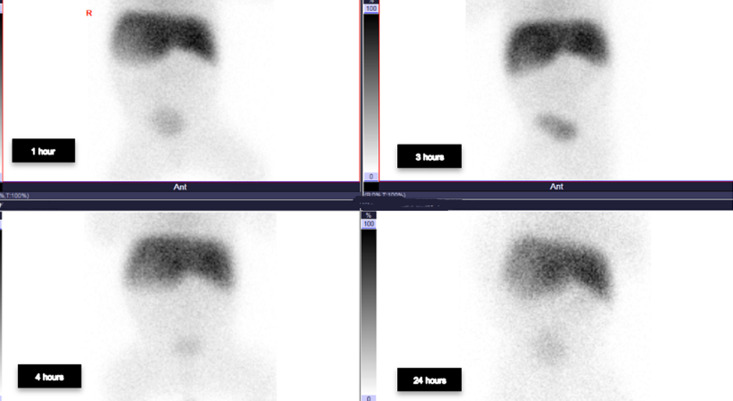
hepatobiliary scintigraphy displayed no definite evidence of biliary tracer excretion in the bowel over 24 hours

**Diagnostic assessment:** chest X-ray showed dextrocardia and cardiomegaly with situs inversus (Figure 1). Abdominal ultrasound (Figure 2) demonstrated hepatomegaly, with the liver occupying both hypochondria, and the gallbladder positioned on the left side. The stomach and spleen were found on the right side, with the spleen measuring 4.2 cm. Hepatobiliary ultrasound (Figure 3) revealed a small, partially contracted gallbladder with irregular walls, suggesting a possibility of biliary atresia. Fish study and chromosomal microarray test were performed to rule out DiGeorge syndrome and other genetic imbalances, both of which were negative. Hepatobiliary scintigraphy (Figure 4) showed no scintigraphic evidence of biliary tracer excretion into the bowel over 24 hours, highly suggestive of biliary atresia. Echocardiogram showed multiple complex congenital heart defects, including dextrocardia, left atrial isomerism, complete atrioventricular septal defect (AVSD), double outlet right ventricle, and severe infundibular narrowing with thickened leaflets and restricted opening of the pulmonary valve. The patient also had a right aortic arch and interrupted inferior vena cava (IVC) draining to the left superior vena cava (LSVC).

**Figure 5:**
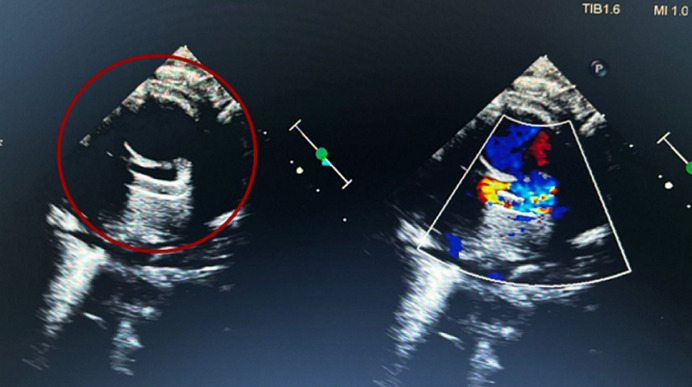
left side on echocardiogram displays the patent ductus arteriosus stenting; the right side shows the flow after the stent placement

**Therapeutic intervention:** the patient initially received respiratory support with CPAP, which was later upgraded to BiPAP, and she was eventually intubated due to persistent respiratory distress. For jaundice management, ursodiol and double phototherapy were initiated. A hepatobiliary scintigraphy scan revealed no biliary tracer excretion into the bowel, leading to a referral for surgical intervention. The patient underwent a laparoscopic Kasai procedure, which was complicated by wound dehiscence, requiring wound exploration, omentectomy, and suturing. Additionally, diagnostic cardiac catheterization was performed along with transcatheter patent ductus arteriosus (PDA) stenting (Figure 5). Nutritional support was provided through nasogastric feeds, and ADEK supplements were administered to address fat-soluble vitamin deficiencies due to disrupted bile flow.

**Follow-up and outcomes:** the patient´s respiratory distress began to resolve, and she was able to maintain oxygen saturation above 75% on room air. Bilirubin levels started trending down following the Kasai procedure (Table 1). Over the next few weeks, the patient progressed well, clinically stable, passing yellow stools, tolerating formula milk every 3 hours, and maintaining oxygen saturation on room air. The parents were counseled on medication administration, red flags, and care instructions and were comfortable being discharged with regular follow-up.

**Table 1:** liver laboratory results of the patient at day 5, day 40 (before Kasai procedure), and day 42 (after Kasai procedure), showing some improvement in bilirubin and alkaline phosphatase levels, while alanine aminotransferase and aspartate aminotransferase remain elevated

	Day 5	Day 40 (before Kasai)	Day 42 (after Kasai)	Normal range
**Total protein**	52.50 gm/L (L)	60.30 gm/L (L)	35.8 (L)	64.00-82.00
**Albumin level**	23.89 gm/L	31.36 gm/L	17.88 (L)	19.00-44.00
**Bili total**	269.50 umol/L (H)	158.20 umol/L (H)	151.70 (H)	3.00-17.00
**Bili total**	269.50 umol/L (H)	136.04 umol/L (H)	-	3.00-17.00
**Bili direct**	54.35 umol/L (H)	131.49 umol/L (H)	-	0.00-15.00
**ALT**	11.00 (H)	114.00	156	
**AST**	44 U/L (H)	254 U/L (H)	401 (H)	15-37
**Alkaline phos**	184.00 IU/L (H)	550.00 IU/L (H)	209	83.00-248.00
**GGT**	510.00 IU/L (H)	376.00 IU/L (H)	-	5.00-354.00

Alt: alanine aminotransferase; AST: aspartate aminotransferase; GGT: gamma-glutamyl transferase

**Patient perspective:** the patient´s mother was satisfied with the therapeutic and management intervention provided by the doctors. She appreciated the collaborative and team effort performed by the different departments in handling her daughter´s case.

**Informed consent:** consent was obtained from the patient´s mother, as the patient is still a minor.

## Discussion

Heterotaxy syndrome, a rare congenital disorder (1: 10,000 births), results from abnormal lateralization of thoracoabdominal organs, often linked to mutations in ZIC3, NODAL, and PITX2 genes critical for embryonic left-right patterning [2,5]. It is classified into left isomerism (e.g, polysplenia) and right isomerism (e.g. asplenia), though overlap exists [1,6]. Cardiovascular malformations occur in 90% of cases, accounting for 4% of all congenital heart diseases and contributing to poor long-term survival [2,6]. Biliary atresia, a severe hepatobiliary disorder (1: 3,000-1: 15,000 births), shares developmental pathways with heterotaxy, particularly in biliary atresia splenic malformation syndrome (BASM), which includes intestinal malrotation and cardiac defects [3,7,8]. Genetic disruptions (e.g. CFC-1) further connect these conditions [7,8]. The prenatal diagnosis remains difficult due to anatomical variability. In our case, postnatal findings (cholestatic jaundice, pale stools, absent biliary excretion on scintigraphy) confirmed the biliary atresia diagnosis. Notably, only 7 of 165 fetuses with heterotaxy in a 2015 study had concurrent biliary atresia [4], highlighting the diagnostic complexity in these cases. Non-cardiac anomalies (e.g. hepatobiliary) are often overshadowed by congenital heart diseases in heterotaxy, since they are commonly presented, delaying the biliary atresia detection in heterotaxy patients [4,9]. The Kasai portoenterostomy is the gold standard for biliary atresia but must be performed before 60 days of life for the best outcomes [3,9]. In heterotaxy, however, anatomical complexity (such as vascular anomalies and situs inversus, etc) and congenital heart diseases can complicate the surgery [9]. Our patient´s timely Kasai procedure aligned with guidelines, yet long-term success rates remain low (30-40% achieve adequate bile drainage) [9,10]. Multidisciplinary care (neonatology, cardiology, surgery) is critical to maintain her stable condition. Despite early Kasai, most patients can progress to end-stage liver disease, in these cases the patient would require a transplantation within the first decade [3,9,10]. A 2019 study by Lim *et al*. emphasized that heterotaxy independently worsens biliary atresia prognosis due to cumulative morbidity complications (e.g. sepsis from asplenia) [9]. Proper and regular antenatal imaging may significantly detect early diagnosis of these complex cases, though sensitivity is limited [4].

## Conclusion

This case report highlights the complex presentation of heterotaxy syndrome in a neonate with severe congenital heart defects and biliary atresia, a rare association that is often overlooked. The successful management of this case underscores the importance of early antenatal diagnosis, detailed fetal imaging, and a multidisciplinary approach. Such strategies are crucial to reducing mortality and morbidity in these highly complex cases.
